# Restoration of Bacterial Microbiome Composition and Diversity Among Treatment Responders in a Phase 2 Trial of RBX2660: An Investigational Microbiome Restoration Therapeutic

**DOI:** 10.1093/ofid/ofz095

**Published:** 2019-04-11

**Authors:** Ken F Blount, William D Shannon, Elena Deych, Courtney Jones

**Affiliations:** 1 Rebiotix, Roseville, Minnesota; 2 BioRankings, St Louis, Missouri

**Keywords:** clinical trial, *Clostridioides difficile* infection, microbiome, microbiota-based therapy, recurrence

## Abstract

**Background:**

RBX2660 is an investigational microbiota restoration therapy in phase 3 clinical development for preventing recurrent *Clostridioides difficile* infections (CDIs). In a randomized, double-blinded placebo-controlled phase 2B trial, RBX2660 was effective at preventing CDI recurrence. The current study was performed to characterize the fecal bacterial microbiome before and after treatment among RBX2660- or placebo-treated responders in that trial.

**Methods:**

Samples were sequenced using 16S methods, and the resulting relative abundance data were fit to a Dirichlet-multinomial distribution to determine group mean relative taxonomic abundance and overdispersion at the class level. Alpha diversity was determined for all samples. Biostatistical tools, including effect size and repeated-measures analysis, were applied to evaluate the statistical significance of observed changes.

**Results:**

At study entry, subjects’ microbiomes were dominated by Gammaproteobacteria and Bacilli, with low abundance of Bacteroidia and Clostridia. After treatment, Bacteroidia, Clostridia, and alpha diversity increased among RBX2660 responders, concomitant with a decrease of Gammaproteobacteria and Bacilli. The resulting compositions differed significantly from baseline compositions, and the changes among RBX2660 responders differed significantly from those in placebo responders, in whom Bacteroidia or Gammaproteobacteria abundance did not change as much. Repeated-measures analyses indicated more rapid and extensive microbiome remodeling among RBX2660 responders compared with placebo responders, and effect size analyses revealed that RBX2660 responders’ microbiomes became more similar to the RBX2660 composition, also compared with placebo responders.

**Conclusions:**

Prevention of recurrent CDI with RBX2660 was associated with restorative microbiome changes that may help resist *C. difficile* colonization and recurrence. RBX2660 was more effective than placebo at restoring participant microbiomes.

## Teaser

In this randomized, double-blinded, placebo-controlled phase 2B trial, prevention of recurrent *Clostridioides difficile* infection with RBX2660 was associated with restorative microbiome changes implicated in resisting *C. difficile* colonization and recurrence. RBX2660 is more effective than placebo at restoring participant microbiomes.

The community of microorganisms resident in the human intestinal tract, or gut microbiome, is increasingly recognized as a key regulator of metabolic and immune homeostasis and a mediator of resistance to some pathogenic infections [1]. Disruption of the composition and/or diversity of the gut microbiome, known as dysbiosis, has been linked with a wealth of disease states [2], and restoration of a “more normal” or “healthier” microbiome is increasingly viewed as a promising treatment option, with a number of experimental microbiome-modulating therapeutics in formal preclinical or clinical development [3–5].

The clinical indication most clearly linked to dysbiosis is recurrent *Clostridioides difficile* infection (CDIs) [6], with clear evidence that restoration of the microbiome can reduce CDI recurrence [7]. RBX2660 is a standardized, stabilized broad-consortium microbiota suspension that is currently in phase 3 clinical development for preventing recurrent CDI ([4, 5]; NCT03244644). In a randomized double-blinded placebo-controlled phase 2B trial, participants who received ≥1 dose of RBX2660 had fewer CDI recurrences than placebo-treated participants 8 weeks after treatment [5].

In this article, we expand on the clinical results of that trial by using a suite of microbiome-appropriate biostatistical tools to demonstrate significant changes to the composition and diversity of RBX2660 responders’ microbiomes after treatment. This shift resulted in increased similarity to the RBX2660 product composition. Participants who responded after placebo treatment showed less microbiome alteration and less convergence toward RBX2660. This analysis underscores the value of RBX2660 as a potential microbiome-restoring therapeutic.

## MATERIALS AND METHODS

### RBX2660 Preparation

RBX2660 delivers a broad consortium of live microbes in a liquid suspension manufactured from the stool of healthy human donors. Donor selection and screening, as well as RBX2660 preparation, were described elsewhere [4], and additional details are provided as Supplementary Material. The blinded part of the phase 2B study included a total of 119 RBX2660 doses from 17 donors. The placebo consisted of normal saline and vehicle solution in the same proportions found in RBX2660.

### Clinical Trial Description

Experimental details and clinical outcomes of the phase 2B trial have been published elsewhere [5] and are provided as Supplementary Material. Briefly, 127 participants with a diagnosis of multirecurrent CDI were enrolled, randomized, and treated in 1 of 3 groups: group A received 2 doses of RBX2660; group B, 2 doses of placebo; and group C, 1 dose of RBX2660 followed by 1 dose of placebo. Successful treatment response was defined as freedom from CDI recurrence at 8 weeks after treatment.

### Sample Collection, Extraction, and Sequencing Analysis

Participants were asked to provide fecal samples before study treatment (baseline) and at 1, 4, and 8 weeks and 6, 12, and 24 months after completion of the assigned blinded study treatment. Participation in the sample collection phase of the trial was optional according to consent requirements; therefore, not all participants were represented in the analysis. To preclude selection bias, we included all received samples from group A, B, or C responders that met the time point criteria of baseline and 10 ± 4, 30 ± 10, or 60 ± 15 days from the date of the last blinded study treatment. No analysis of samples outside these ranges has been conducted. Fecal samples from nonresponders were not included in this analysis because all were treated with open-label RBX2660 after determination of treatment failure, complicating quantitative comparison with other sample groups. An aliquot of each RBX2660 dose administered in the blinded phase of the study was also included in sequencing analyses. All samples were sequenced using 16S methods adapted from those developed for the National Institutes of Health Human Microbiome Project [8] and conducted by Diversigen. 16S ribosomal RNA gene sequences were clustered into operational taxonomic units (OTUs) at a similarity cutoff value of 97%, using the UPARSE algorithm [9]. Additional details are provided as Supplementary Material.

### Determination of Taxonomic Abundance and Diversity Measures

Relative taxonomic abundance at the class level was determined for all samples based on OTU data. Group mean relative abundance values were determined by fitting sample relative abundance values to a Dirichlet-multinomial (DM) distribution using a maximum likelihood method, as described elsewhere [10], to yield 2 summary statistics: pi (π), the vector of mean taxa proportions in a set of samples, and theta (θ), a measure of overdispersion, or sample variation between samples that is independent of sequencing depth or sample size. Alpha diversity was calculated as the Shannon and Simpson indices [11].

### Multidimensional Scaling Analysis

Multidimensional scaling (MDS) analysis was used to map all individual samples onto 2-dimensional space with a Bray-Curtis dissimilarity measure [10]. The mean taxonomic composition for each time and treatment group, estimated by the DM parameter π, was included as an additional sample in the MDS analysis.

### Statistical Analyses

All statistical analyses were performed at the class taxonomic level, except where noted, collapsing taxa that contribute <1% cumulatively into a single taxon (“other”). The statistical significance of intergroup differences was determined for π using a Wald-type test and for θ using a Likelihood ratio test [10]. The statistical significance of π and θ differences among patient-matched longitudinal samples (eg, samples from the same subject at different time points) were determined using a permutation test [12]. Hypothesis testing for repeated-measures analysis was performed using the repeatDM algorithm [13]. Average alpha diversity indices were compared between groups using a univariate Wilcoxon test.

### Effect Size Analysis

Effect size (ES) for microbiome comparisons was calculated as a modified Cramer criterion ϕ, as described elsewhere [10], with larger ϕ indicating a larger difference in microbiome taxa distributions.

## RESULTS

### Clinical Trial Results and Participant Fecal Samples

As described elsewhere in an interim analysis [5], 25 of 41 participants in group A (2 RBX2660 doses) and 25 of 42 in group C (1 RBX2660 and 1 placebo dose) responded to treatment, for a response rate of 60% among all participants who received ≥1 active treatment, compared with a 43% response rate among participants who received only placebo (group B, 19 of 44). Treatment response did not differ significantly between participants receiving 1 and those receiving 2 RBX2660 doses (groups A and C; *P* > .05). Among 69 responders, 58 provided ≥1 fecal sample which was included in this analysis (Tables 1 and 2). There was no evidence of a significant difference among treatment groups with respect to age, sex, prior episodes, duration of enrolling episode, or antibiotic administered for the enrolling episode (*P* > .05; Kruskal-Wallis test [χ^2^ text for sex]). Fecal samples from nonresponders were not included in this analysis because all were treated with open-label RBX2660 after determination of treatment failure, confounding quantitative comparison to other sample groups.

**Table 1.** Demographics of Participants in Current Analysis^a^

Characteristic	Group A (n = 22)	Group B (n = 15)	Group C (n = 21)
Age, median (range), y	63 (24–88)	65 (19–90)	58 (26–88)
Female sex, no. (%)	13 (59)	12 (73)	14 (64)
White race, no. (%)	22 (100)	15 (93)	22 (100)
Antibiotic used at screening, no. (%)			
Vancomycin	20 (91)	13 (87)	19 (90)
Fidaxomicin	1 (4.5)	1 (6)	1 (5)
Metronidazole	1 (4.5)	0 (0)	0 (0)
Vancomycin + other	0 (0)	1 (7)	1 (5)
No. of CDI episodes, median (range)	3 (3–7)	3 (2–5)	4 (3–10)
Duration of CDI episodes, median (range), d	13 (2–64)	17 (3–47)	14 (2–51)

Abbreviation: CDI, *Clostridioides difficile* infection.

^a^Group A received 2 doses of RBX2660; group B, 2 doses of placebo; and group C, 1 dose of RBX2660 followed by 1 dose of placebo. There was no evidence of significant differences among treatment groups with respect to age, sex, prior episodes, duration of enrolling episode, or antibiotic administered for the enrolling episode (Kruskal-Wallis test).

**Table 2.** Samples Included in Current Analysis

Treatment Group^a^	Treatment Responders, No.		Samples, No.				
	Total	Included in Analysis	Baseline	10 d	30 d	60 d	RBX2660^b^
A	25	22	18	18	17	13	44
B	19	15	13	11	10	12	
C	25	21	18	17	13	18	21

^a^Group A received 2 doses of RBX2660; group B, 2 doses of placebo; and group C, 1 dose of RBX2660 followed by 1 dose of placebo.

^b^Denotes RBX2660 dose samples included in sequencing analyses.

### MDS Analysis: Responder Microbiomes Became More Similar to the RBX2660 Composition After Treatment

**Figure 1.**
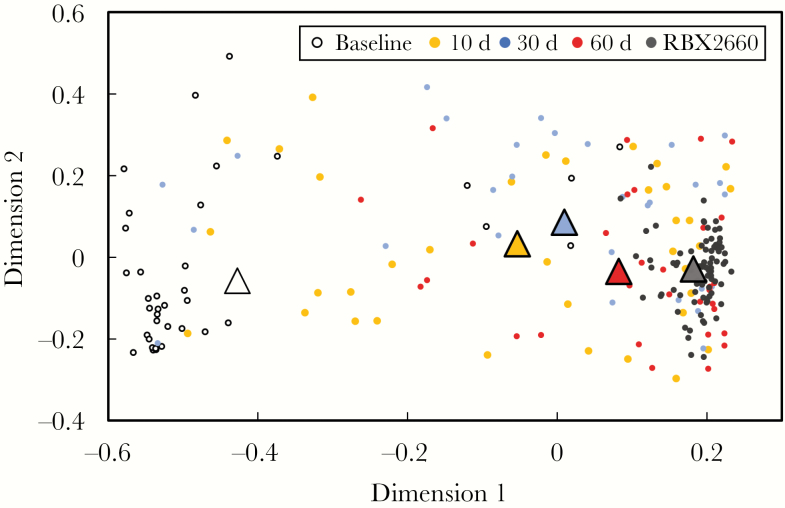
Multidimensional scaling analysis based on Bray-Curtis dissimilarity for RBX2660 product samples and RBX2660 responder samples before treatment (baseline) and 10, 30, and 60 days after treatment. The mean microbiome composition for each time point group (*triangles*) was calculated based on least-squares fit to a Dirichlet multinomial and was included in the analysis.

The MDS analysis indicated that participant microbiomes were dissimilar to RBX2660 microbiota at baseline and not clustered as closely together as RBX2660 samples (Figure 1). After treatment, RBX2660 responders’ microbiomes were more similar to RBX2660, while retaining heterogeneity within each time point group, as indicated by the spread of the samples within those time points relative to the spread of the RBX product samples. The group mean taxonomic compositions for each time group were included in the MDS analysis and also showed a clear progression to be more similar to RBX2660 after treatment (triangles in Figure 1). Importantly, there were no apparent differences between RBX2660 treatment groups A and C at any time point (Supplementary Figure 1).

### Effect of RBX2660 Treatment on the Taxonomic Composition of Participant Microbiomes

**Figure 2.**
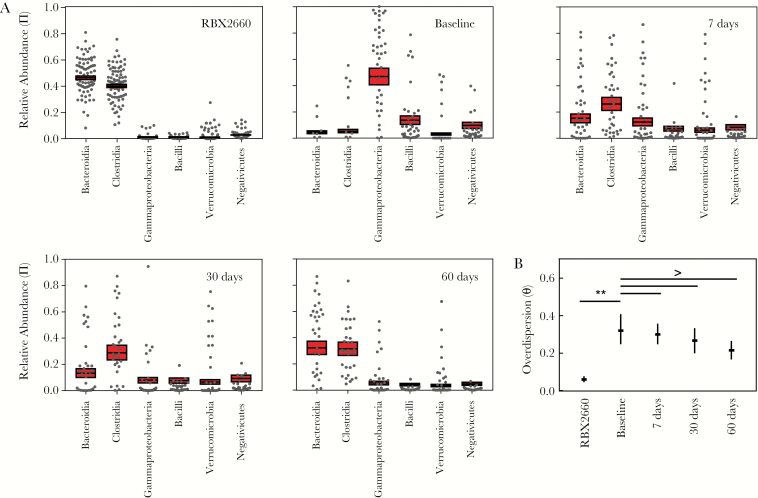
Sample and group mean taxonomic compositions among RBX2660 product samples and responder samples before treatment and at 10, 30, and 60 days after treatment. *A,* Relative abundance of taxonomic classes present at ≥5% abundance, including Bacteroidia, Clostridia, Gammaproteobacteria, Bacilli, Verrucomicrobia, and Negativicutes. Individual samples are represented as dots, and group means (π) with upper and lower confidence intervals (*red boxes*) were calculated based on maximum likelihood estimate using the Dirichlet multinomial. *B,* Overdispersion (θ) at the taxonomic class level for sample time point groups, shown as medians with upper and lower confidence intervals calculated using the method of moments. Comparisons among time groups are noted as significant (*P* < .05) or not significant (NS).

Relative abundance values at the taxonomic class level were calculated from OTU data for all individual participant samples and RBX2660 doses administered in this study (Figure 2A). Among the RBX2660 doses administered, Bacteroidia and Clostridia were the predominant classes with generally low abundance of other classes including Gammaproteobacteria and Bacilli. This finding is generally consistent with reported compositions for healthy North American or European cohort studies [8, 14], as would be expected because RBX2660 is sourced from regularly screened healthy human donors. There was some variability in composition from dose to dose, but a previous analysis found no evidence of efficacy differences among these doses or donors [15].

Visual inspection of the data indicated that among baseline (pretreatment) samples Gammaproteobacteria and Bacilli were the predominant classes with low abundance of other classes, including Clostridia and Bacteroidia (Figure 2A), consistent with published characterizations of recurrent CDI patient microbiomes [16, 17]. After treatment, participant microbiomes became progressively more like RBX2660, with increasing predominance of Clostridia and Bacteroidia and decreasing Gammaproteobacteria and Bacilli (Figure 2A). The relative distribution of orders and families within each of these classes remained mostly stable throughout the time course (Supplementary Figure 2.1–2.4). Notably, Enterobacteriales vastly predominated among Gammaproteobacteria; Lachnospiraceae and Ruminococcaceae predominated among Clostridia; Lactobacillaceae seemed to decrease in prominence after treatment among Bacilli; and Bacteroidia comprised relatively even predominance of Bacteroidaceae, Prevotellaceae, Rikenellaceae, and Porphyromonadaceae.

To facilitate statistical hypothesis testing, summary statistics were determined for each time and treatment group by fitting the data to a DM via maximum likelihood estimation [10] to derive group mean relative abundance (π) and overdispersion (θ), which is an expression of between-sample variation within a group. The DM is particularly appropriate for summarizing microbiome data because it facilitates direct hypothesis testing among treatment groups while accounting for compositional interdependence—that is, an increase in the relative abundance of 1 taxon necessarily accompanies a decrease in at least 1 other taxon. Before comparing time groups, we confirmed that there were no significant differences between data for treatment groups A and C data at each time point (*P* > .05; generalized Wald-type test; Supplementary Table 1), supporting the use of the pooled data set for subsequent analyses.

Based on a permutation test, mean participant microbiome compositions at the class level (π) differed significantly at all time points after RBX2660 treatment compared with the baseline composition (Figure 2A and Table 3). Baseline and posttreatment groups also had higher overdispersion (θ) at the class level than RBX2660 (*P* < .05; Figure 2B), indicating higher variation. This was expected because RBX2660 is manufactured through a consistent, quality-controlled process to minimize variation. Although θ for visually trended lower after treatment compared with baseline, these differences were not statistically significant (Figure 2B). When calculated at lower taxonomic levels, posttreatment and baseline θ values were significantly different (Supplementary Table 2).

**Table 3.** Significance of Differences in Group Mean Relative Abundance (π) Between Time Points, Determined Using a Permutation Test

Time Point	*P* Value by Time Point			
	Baseline	10 d	30 d	60 d
Baseline	…	.001	.001	.001
10 d	.001	…	.12	.006
30 d	.001	.12	…	.03
60 d	.001	.006	.03	…

### Effect of RBX2660 Treatment on Alpha Diversity of Participant Microbiomes

**Figure 3.**
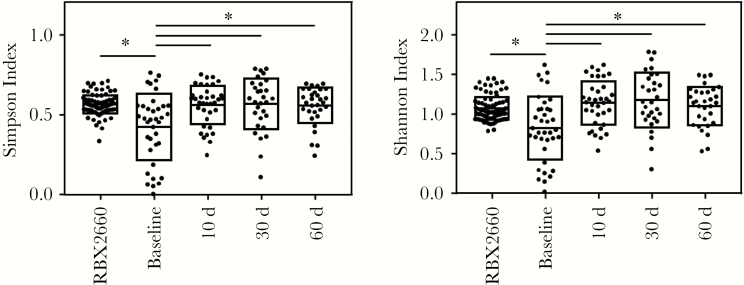
Alpha diversity of RBX2660 and participant samples for each time point group, expressed as the Simpson or Shannon index. Boxes represent group means with standard deviations. *P < . 05 (Wilcoxon test).

Alpha diversity is commonly expressed as the Shannon index, an unweighted measure, or the Simpson index, a measure weighted by relative abundance [18]. Both indices were significantly increased after RBX2660 treatment compared with baseline (Figure 3; Wilcoxon test), consistent with findings of prior analyses of recurrent CDI [17], and all posttreatment groups had alpha diversity similar to that of RBX2660.

### ES Analysis: Convergence of Posttreatment Microbiomes Toward the RBX2660 Profile

**Figure 4.**
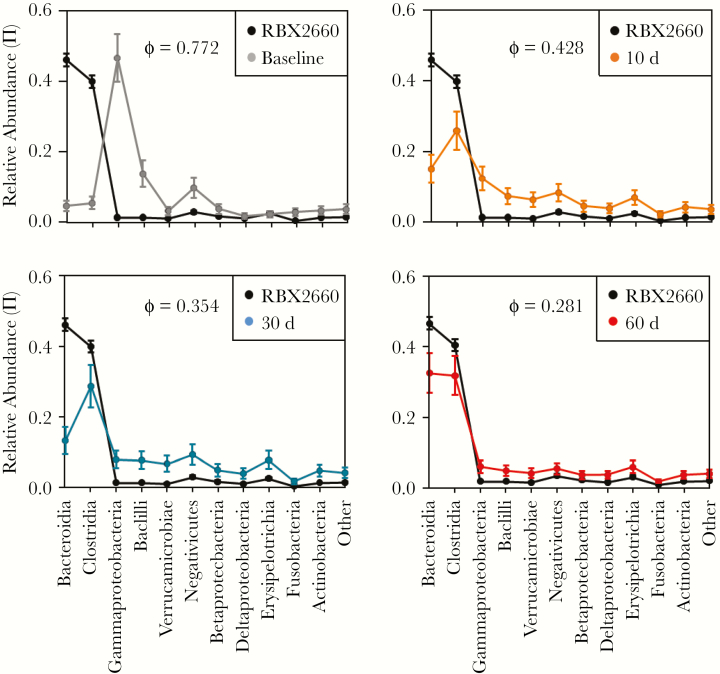
Effect size of each time point group compared with RBX2660. Mean relative abundance vectors (π) at the taxonomic class level are shown with upper and lower confidence intervals as determined by fitting to a Dirichlet-multinomial distribution. Effect size is expressed as a modified Cramer criterion, ϕ, for each pairwise comparison.

As a broad-consortium treatment sourced from healthy human donors and with a composition similar to healthy cohorts, RBX2660 can be considered 1 representative of a healthier microbiome. Therefore, as a measure of the effectiveness of RBX2660 treatment, we quantified the extent to which treatment shifted participants’ microbiome composition toward the RBX2660 composition. ES, expressed as a modified Cramer criterion (ϕ), is a method by which the similarity or dissimilarity between 2 microbiome sample populations can be quantified [10, 19]. At baseline, ES for the comparison with RBX2660 was large (ϕ = 0.703), with apparent differences in Bacteroidia, Clostridia, Gammaproteobacteria, Bacilli, and Negativicutes (Figure 4). At 10, 30, and 60 days after treatment, ϕ decreased to 0.424, 0.294, and 0.261, respectively, confirming progression of participants’ microbiomes to more closely resemble RBX2660.

### Differences in Microbiome Changes and Restoration Among Placebo-Treated Responders

**Figure 5.**
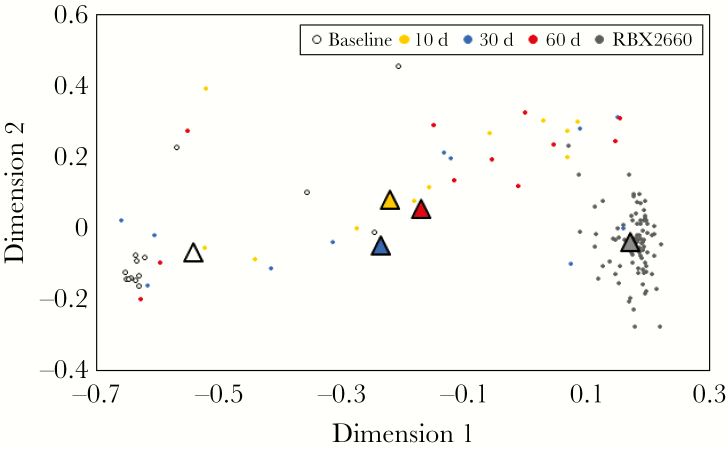
Multidimensional scaling analysis based on Bray-Curtis dissimilarity for RBX2660 product samples and placebo responder samples before treatment (baseline) and 10, 30, and 60 days after treatment. The mean microbiome composition for each time point group (*triangles*) was calculated based on Dirichlet-multinomial distribution and was included in the analysis.

**Figure 6.**
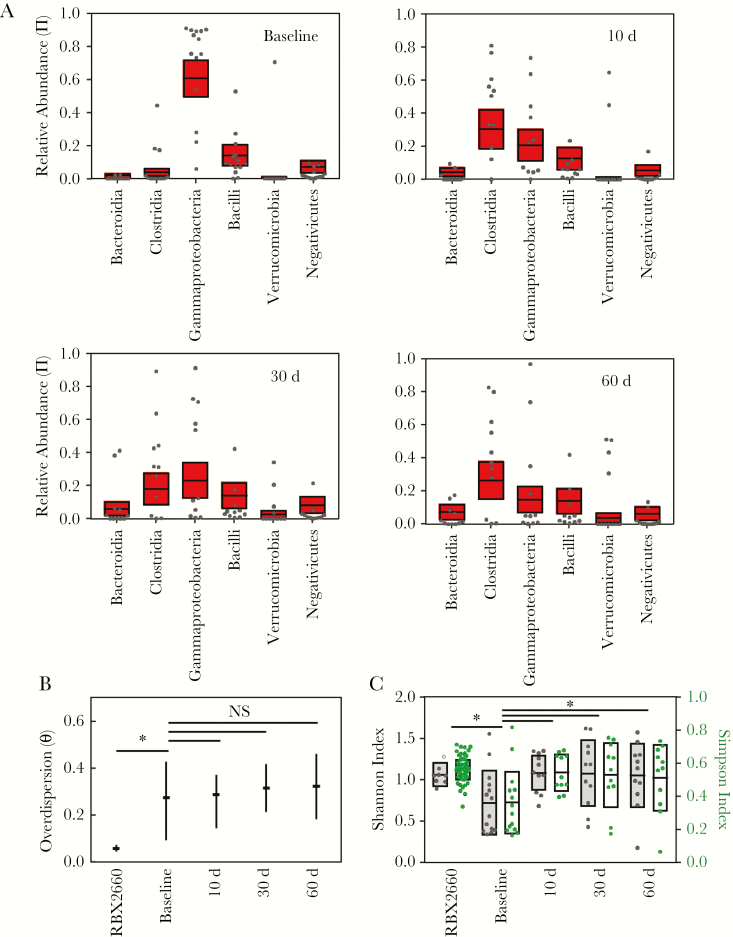
Sample and group mean taxonomic compositions and diversity among RBX2660 product samples and placebo responder samples before treatment (baseline) and 10, 30, and 60 days after treatment. *A,* Relative abundance of taxonomic classes present at ≥5% abundance, including Bacteroidia, Clostridia, Gammaproteobacteria, Bacilli, Verrucomicrobia, and Negativicutes. Individual samples are represented as dots, and group means (π) with upper and lower confidence intervals (*red boxes*) were calculated based on maximum likelihood estimate using the Dirichlet multinomial. *B,* Overdispersion (θ) for sample groups, shown as median with upper and lower confidence intervals calculated using the method of moments. Comparisons among time groups are noted as significant (*P < .05) or not significant (NS). *C,* Alpha diversity for individuals and sample groups, expressed as the Simpson or Shannon index. Boxes represent group means with standard deviations. *P < .05 (Wilcoxon test).

Visual inspection of a Bray-Curtis MDS analysis indicated that the microbiomes among placebo-treated responders diverged from baseline to a lesser extent than those in RBX2660-treated participants and did not converge with RBX2660 (Figure 5). As well, the mean compositions (π) at the class level changed less after placebo treatment than after RBX2660 (Figure 6A). Most notably, Bacteroidia never exceeded 15% abundance and did not differ significantly from baseline at any time point after placebo treatment (*P* > .05; Wilcoxon test). Likewise, Gammaproteobacteria remained a significant fraction of placebo responders’ microbiomes even 60 days after treatment. The overall composition was significantly different between placebo responders and RBX2660 responders 60 days after treatment (*P* = .02; Wald-type test; Table 4), confirming that RBX2660 treatment is more effective at shifting the microbiome composition. Overdispersion (θ) among placebo responders trended higher after treatment (Figure 6B) and was significantly higher than among RBX2660 responders at 30 and 60 days after treatment (*P* < .05; likelihood ratio test). Finally, ES analysis confirmed that placebo-treated responders’ microbiomes did not shift as close to RBX2660 after treatment, compared with RBX2660-treated responders (ϕ = 0.36, 0.45, and 0.36 at 10, 30, and 60 days, respectively; Supplementary Figure 3).

**Table 4.** Comparisons of Group Mean Relative Abundance (π) and Overdispersion (θ) Between RBX2660 Responders and Placebo Responders by Time Point^a^

Time Point	*P* Value	
	π	θ
Baseline	.99	.16
10 d	.93	.76
30 d	.06	.02
60 d	.02	<.01

^a^The π values were compared using Wald-type tests, and the θ values using likelihood ratio tests.

### Differences in Dynamics of Microbiome Changes Between RBX2660- and Placebo-Treated Responders

**Figure 7.**
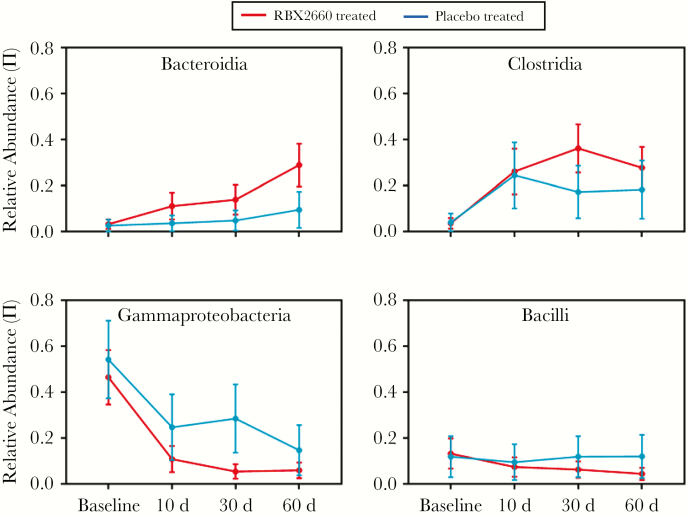
Relative abundance of key taxonomic classes at baseline and posttreatment for participant samples included in the repeated-measures analysis. Mean relative abundance (π) values with confidence intervals are shown for RB2660- and placebo-treated participants.

To assess longitudinal changes among patient-matches samples, we performed repeated-measures analysis on the subset of samples from RBX2660- or placebo-treated participants from whom all 4 time points were available (baseline and 10, 30, and 60 days after treatment; 16 RBX2660-treated and 7 placebo-treated participants). With use of DMRepeat, which adjusts for within-subject correlation [13], the longitudinal microbiome changes differed significantly between RBX2660 and placebo responders (Figure 7 and Supplementary Figure 4; *P* < .001). Notable among these differences were a greater increase in Bacteroidia and decrease in Gammaproteobacteria among RBX2660 responders at 10 days.

## DISCUSSION

A disrupted intestinal microbiome is presumed to contribute to many human diseases, including recurrent CDI. Accordingly, therapeutics that restore the microbiome could become an effective health care strategy. The process of developing them will benefit from the statistical demonstration of changes in patients’ microbiomes that are associated with improvement in the disease state. Here we report microbiome changes associated with a randomized, double-blind, placebo-controlled trial of the investigational microbiome restoration therapeutic RBX2660. We also present and apply a microbiome biostatistics tool set to evaluate the statistical significance of treatment-associated changes.

The microbiome of our participants before RBX2660 or placebo treatment (baseline) is consistent with what is described in the literature. The taxonomic classification levels at which data are reported varies among publications, so characterization at the class level is inferred for comparison. The majority of our participants received vancomycin or metronidazole—the most commonly prescribed CDI antibiotics at the time of the trial. Both antibiotics are known to profoundly alter microbiota, including reducing Bacteroidia and Clostridia and facilitating overgrowth of Gammaproteobacteria [20, 21]. Both are also known to decrease alpha diversity. These disruptions leave patients susceptible to recolonization and CDI recurrence. Our participants had similar microbiota alterations at baseline, confirming that they are reasonably representative of a general recurrent CDI population.

Only 3 participants received fidaxomicin before study treatment. Fidaxomicin was developed as a more selective agent that causes less microbiome disruption and thereby reduces recurrence rates at 30 days after treatment [22]. Compared with vancomycin, fidaxomicin and other newer antibiotics currently being developed are generally more sparing of Bacteroidia and Clostridia [23–28]. Although these newer agents have shown promise in clinical trials, Gammaproteobacteria overgrowth and other microbiome disruptions can persist [29, 30], which we observed among fidaxomicin-treated patients at baseline (Supplementary Table 3). Thus, the literature and our data underscore that there remains a need for microbiome-restoring therapies.

The approach most extensively associated with restoring the microbiome and reducing recurrence after CDI treatment is fecal microbiota transplant (FMT), although methods, donor sources, and outcomes vary among reports [31]. The increased Bacteroidia, Clostridia, and alpha diversity and decreased Gammaproteobacteria and Bacilli after RBX2660 treatment have also been observed after FMT [7, 32]. There is some evidence in the literature that varying FMT compositions result in different posttreatment microbiome compositions. For example, the relative abundance of Bacteroidia in FMT compositions varied from <10% to >50% between 2 studies, and posttreatment patient microbiomes varied accordingly [7, 33]. If large enough, these types of variations conceivably could lead to differences in effectiveness. Moreover, because preparation, storage, and delivery methods can affect the composition or viability of FMT [34], there is a clear need to standardize the process and product and implement quality control measures, as has been done for RBX2660.

There is 1 other report of microbiome changes after treatment with a standardized investigational microbiome therapeutic undergoing formal clinical evaluation for recurrent CDI. SER-109 is a human stool–sourced, spore-based formulation that demonstrated promising efficacy in an exploratory dose-ranging trial [3]. Although this benefit remains to be demonstrated in a placebo-controlled trial, participant microbiomes showed similar restoration as RBX2660 responders—regrowth of Clostridia, reduction of Enterobacteriaceae (within Gammaproteobacteria class), and increased alpha diversity by 4 weeks after treatment. Bacteroidia regrowth after SER-109 was not as extensive as after RBX2660 treatment, with only 11 of 29 participants showing an increase. This may be due to a lack of Bacteroidia in the SER-109 product; placebo-controlled clinical trials will be needed to determine the importance of this difference to clinical efficacy.

The key differences between RBX2660- and placebo-treated responders’ microbiomes after treatment may reveal why RBX2660 showed a superior clinical response. Microbiome changes after placebo treatment can be considered a reasonable representative of the general population with postantibiotic recurrent CDI, because the placebo response rate mirrors known postantibiotic recurrence-free rates [35] and the placebo contained no microbes. Although some changes were observed among placebo responders, RBX2660 showed more rapid and extensive restoration of Bacteroidia and Clostridia and decreased Gammaproteobacteria and Bacilli. This suggests there may be a minimal level of restoration that is essential shortly after antibiotic treatment if recurrence prevention is to be effective. It may be that key metabolic functions, competitive ecology, decreased inflammatory provocation and colonization resistance are critical during this period when patients are most vulnerable to recurrence. Future studies could aim to address this by varying the dose level or compositions of microbiome treatments and by performing repeated-measures analysis of aggregate or larger cohorts.

Some reports have suggested that direct and durable engraftment of FMT-derived strains into patients is important to effectiveness [16]. However, given the severe dysbiosis among patients with recurrent CDI, it may be more important to rapidly instill and restore a “healthier” or “more normal” microbiome than to directly and durably engraft. Once protected from recurrence by short-term restoration, patients could equilibrate to their normal microbiome as guided by diet, environment, lifestyle, and other factors, such that durable retention of FMT strains is not needed. One study at least supports this model, in that restoration of key functions, rather than complete donor-to-patient engraftment, was important to effectiveness [7]. Stated differently, instilling a healthy microbiome may dislodge patients from a localized minimum of dysbiosis, allowing reequilibration to their healthier composition.

If this model is correct, it is useful to consider what characteristics of a healthier, more normal microbiome could accomplish this, recognizing that there is no reference-standard healthy microbiome composition. The Human Microbiome Project [8], Meta-HIT [14], and other healthy cohort studies [36] all found Bacteroidia and Clostridia to be prevalent, which makes sense given their association with colonization resistance [37, 38]. They also found lower abundance of Gammaproteobacteria (particularly Enterobacteriaceae), which are commonly associated with susceptibility to *C. difficile* colonization, gastrointestinal inflammation, and other disorders [39, 40]. Finally, higher alpha diversity is also associated with microbiome health [17]. Although it is hazardous to overgeneralize these characteristics, they are represented in many examples of healthy microbiota, including RBX2660, which is manufactured from stool donations of generally healthy individuals. Our results showed rapidly increasing similarity to RBX2660 after treatment, which supports a restoration model for the clinical efficacy of RBX2660. Future studies will seek to explore this more deeply by monitoring microbiome compositions longer after treatment and identifying key functionally restorative elements of RBX2660.

## Supplementary Data

Supplementary materials are available at *Open Forum Infectious Diseases* online. Consisting of data provided by the authors to benefit the reader, the posted materials are not copyedited and are the sole responsibility of the authors, so questions or comments should be addressed to the corresponding author.

Supplementary_Material
