# Supplementary material for: Restoration of Bacterial Microbiome Composition and Diversity Among Treatment Responders in a Phase 2 Trial of RBX2660: An Investigational Microbiome Restoration Therapeutic
Source: Open Forum Infect Dis. 2019 Apr 11;6(4):ofz095. doi: 10.1093/ofid/ofz095 (PMC6475591; doi:10.1093/ofid/ofz095)
Supplement: Supplementary_Material [file ofz095_suppl_supplementary_material.docx]

SUPPLEMENTARY INFORMATION

RESTORATION OF BACTERIAL MICROBIOME COMPOSITION AND DIVERSITY AMONG TREATMENT RESPONDERS IN A PHASE 2 TRIAL OF RBX2660—AN INVESTIGATIONAL MICROBIOME RESTORATION THERAPEUTIC

Ken F Blount^1*^, William D. Shannon^2^, Elena Deych^2^, Courtney Jones^1^

^1^Rebiotix Inc, Roseville, MN USA; ^2^BioRankings LLC, St. Louis, MO USA;

^*^To whom correspondence should be addressed

**Supplementary methods**

***RBX2660 preparation***

RBX2660 is manufactured as single-dose, ready-to-use units, each in an enema bag containing a 150 mL suspension of ≥10^7^ live organisms/mL. Each dose has a unique identifier that allows traceability from donor to donation to manufactured batch. RBX2660 doses were stored frozen at −80°C in a secure location at the manufacturer. As needed, RBX2660 doses were shipped frozen to the site in a temperature-controlled container, thawed in a refrigerator for 24 hours, and administered within 48 hours after thawing.

***Clinical Trial Description***

A complete description of the Phase 2B clinical trials has been published previously [1]. Briefly, 127 eligible and consenting participants at least 18 years old with a diagnosis of multi-recurrent CDI were enrolled, randomized, and treated in one of three treatment groups: two doses of RBX2660 (Group A), two doses of placebo (Group B), or one dose of RBX2660 followed by one dose of placebo (Group C). Participants were required to complete antibiotic treatment for CDI with resolution of symptoms. Administration of the first study dose commenced 24-48 hours following completion of antibiotics, with the second study dose administered 7 ± 2 days thereafter. Successful treatment response was defined as freedom from CDI recurrence at 8 weeks following treatment, and treatment failure was defined as meeting all following criteria at <8 weeks after completion of both assigned blinded study treatments: diarrhea, a positive laboratory diagnosis for *C. difficile* or its toxins as conducted and reported by the study investigator, a need for retreatment for CDI, and no other cause for CDI symptoms.

The efficacy for Groups A, B, and C were 61%, 45%, and 67%, respectively. The primary endpoint was not met (Group A versus Group B; *p*=.152). One 2660 dose was superior to placebo (Group C versus Group B, *p*=.048). Two doses of RBX2660 had equivalent efficacy to one dose (Group C versus Group A, *p*=.589). The proportion of adverse or serious adverse events did not differ significantly among blinded treatment groups.

***Sample collection, extraction, and sequencing analysis***

Participants were assigned a unique subject ID number which allowed anonymized traceability of collected samples and data. Participants were asked to provide fecal samples at the time of screening prior to study treatment (baseline) and at 1, 4, and 8 weeks and 6, 12, and 24 months after completion of the assigned blinded study treatment, with samples collected at home using kits provided by the study team which were pre-labeled with the subject identifier. Each kit included sample collection instructions, a collection container with sealable lid, disposable scaffolding, secondary container closure bag, icepack, and insulated shipping container with a prepaid overnight shipping label. No stabilizers were added at the time of collection or thereafter. Once received at our location, samples were immediately aliquoted into small volumes, labeled with the subject identifier and date received, and stored at -80C for later analysis. For each sample, the time interval between treatment and sampling was determined and recorded once treatment information was unblinded.

Participation in the sample collection phase of the trial was optional per consent requirements; therefore, not all participants were represented in the analysis. To preclude selection bias, we included all received samples from Group A, B, or C responders which met time point criteria of baseline and 10 ± 4, 30 ± 10, or 60 ± 15 days from the date of the last blinded study treatment. Fecal samples from non-responders were not included in this analysis because all were treated with open-label RBX2660 upon failure determination (median time of 7 days from last blinded treatment to failure determination). This additional treatment could have induced additional microbiome changes, complicating statistical comparisons to baseline samples. An aliquot of each RBX2660 batch administered to any participants was also included in the sequencing analysis.

16S rRNA gene sequencing methods were adapted from those developed for the National Institutes of Health Human Microbiome Project [2] and conducted by Diversigen, Inc (Houston, TX). Briefly, bacterial genomic DNA was extracted using MO BIO PowerSoil DNA Isolation Kit (MO BIO Laboratories). The 16S rDNA V4 region was amplified by PCR and sequenced in the MiSeq platform (Illumina) using the 2x250 base-pair paired-end protocol yielding pair-end reads that overlap almost completely. The primers used for amplification contain adapters for MiSeq sequencing and single­end barcodes which allow pooling and direct sequencing of PCR products.

The 16S rRNA gene pipeline data incorporated phylogenetic and alignment-based approaches to maximize data resolution. Read pairs were demultiplexed based on unique molecular barcodes, and reads were merged using USEARCH v7.0.1090 [3], allowing zero mismatches and a minimum overlap of 50 bases. Merged reads were trimmed at first base with Q5, and a quality filter was applied to the resulting merged reads, with reads containing above 0.05 expected errors discarded. 16S rRNA gene sequences were clustered into Operational Taxonomic Units (OTUs) at a similarity cutoff value of 97% using the UPARSE algorithm[4]. OTUs were mapped to an optimized version of the SILVA Database[5] containing only the 16S v4 region to determine taxonomies. Abundances were recovered by mapping the demultiplexed reads to the UPARSE OTUs. A custom script constructed a rarefied OTU table from the output files generated in the previous two steps for downstream analyses.

All statistical analyses were performed at the Class taxonomic level, collapsing rare taxa that contribute less than 1% cumulatively into a single taxon (“Other”).

**Taxa Relative Abundances and Overdispersion**

Reducing a set of microbiome taxa count samples into a summary statistic, such as the proportion or relative abundances of each taxa and the amount of variability across samples, allows researchers to examine the data much as they would with a mean and standard deviation from normally distributed data. Microbiome data follows a Dirichlet-multinomial distribution which has 2 parameters: π (pi) which is the vector of expected taxa proportions in a set of samples, and θ (theta) which is a measure of variability called overdispersion which is the natural sample-to-sample variability that does not disappear with increased read numbers[6]. Both π and θ are sufficient statistics and can be estimated using the maximum likelihood (ML) or method of moments (MOM) algorithms.

With this model *p* values for intergroup comparisons can be calculated directly through Wald-type statistics when the samples are independent, or permutation tests when the samples are dependent (e.g., samples from the same subject at different time points. The Wald-type test has been detailed previously[6] and beyond the scope of this paper. The permutation test is done by randomly shuffling the samples across time points within a subject, calculating the likelihood ratio statistic from the DM model, and using this as the null distribution of no difference in groups. The permutation test compares two time points at a time. This is a standard permutation technique described previously[7].

***Multidimensional Scaling Analysis (MDS)***

Multidimensional scaling (MDS) is a visual technique to assess the similarity of individual samples that is based on their pairwise sample distances[8]. The purpose of MDS is to find a set of points on a low dimension plot, say 2 dimensions, such that each sample is assigned to one point on the plot, on the pairwise distances among those projected points is associated to the actual distances calculated from the raw data. Variations in how the associated is calculated are technically beyond the scope of this paper but are based on some measure of error between the observed and the projected pairwise distances (classical and metric MDS), or on a linear model between the observed and the projected pairwise distances (nonmetric MDS). An efficient MDS algorithm will find the set of projected points such that the selected association is maximum.

In this analysis the Bray-Curtis distance between all pairs of samples based on sample taxa proportions was calculated[9]. Non-metric MDS was performed, mapping all samples onto a two-dimensional space for visualization. The MDS coordinates for all samples were plotted where the closer two samples are on the plot, the more similar are those samples taxa. In addition to the samples each group’s mean taxa estimated by the DM π parameter is plotted and represented as triangles. Each sample and group π are color coded by type (patient or RBX2660) and time (baseline, 7 days, 30 days, 60 days), and group π’s (triangles).

**Effect Size Analysis**

To assess the shift in patients’ microbiome towards RBX2660, we calculated the effect size (ES) of group difference and show the patient’s microbiome gets closer to RBX2660 microbiome over time. An ES is a measure of how far apart two means are from each other and is used in power and sample size calculations in experimental design. In this paper the ES is a measure of the difference between the average microbiome taxa abundances of two populations with the bigger the difference the larger the effect size. Technical details on the microbiome ES, a Modified Cramer's Phi Criterion ϕ, and how it is used to design studies has been previously described[6, 7].

**Alpha Diversity**

Alpha-diversity is a measure of how many different types and relative abundances of taxa there are within a sample[9]. Two diversity indices used here are the Shannon and Simpson measures. Shannon diversity is a measure of entropy or information. When the taxa in a sample are present in equal abundance the Shannon diversity is maximum and gets smaller as relative abundances start varying for different taxa. Simpson diversity is the probability that two taxa picked at random from a sample are the same. This is maximized at 1 when there is a single taxon, and smallest when all taxa are present in equal proportions.

Since alpha-diversity is measured on single samples, comparisons of the diversity indices of each sample across groups is done using a univariate statistical test (e.g., Wilcoxon). This will test if the average diversity index is the same or different between groups but does not indicate if the spread or variability of the diversity measures calculated for each sample is the same or different.

**Overdispersion as beta-Diversity**

Beta-diversity measures variation between samples in a population[9]. This is usually calculated from the similarity or dissimilarity of every pair of microbiome samples. One measure is the Bray-Curtis distance matrix. All pairwise distances are then averaged to quantify the population beta-diversity but ignore the variability and lack of independence of these values.

The Dirichlet-multinomial θ (theta) parameter is a beta-diversity index calculated as a single number over all of the microbiome samples. This is analogous to the standard deviation from a set of normally distributed variables. It has the advantage of being calculated on the taxa count data and does not require that samples be summarized first by pairwise distances. Larger values of θ indicate samples are more dissimilar from each other, while small values of θ indicate more sample similarity.

**Supplementary Figure S1**. MDS analyses of Group A compared to Group C at each time point.


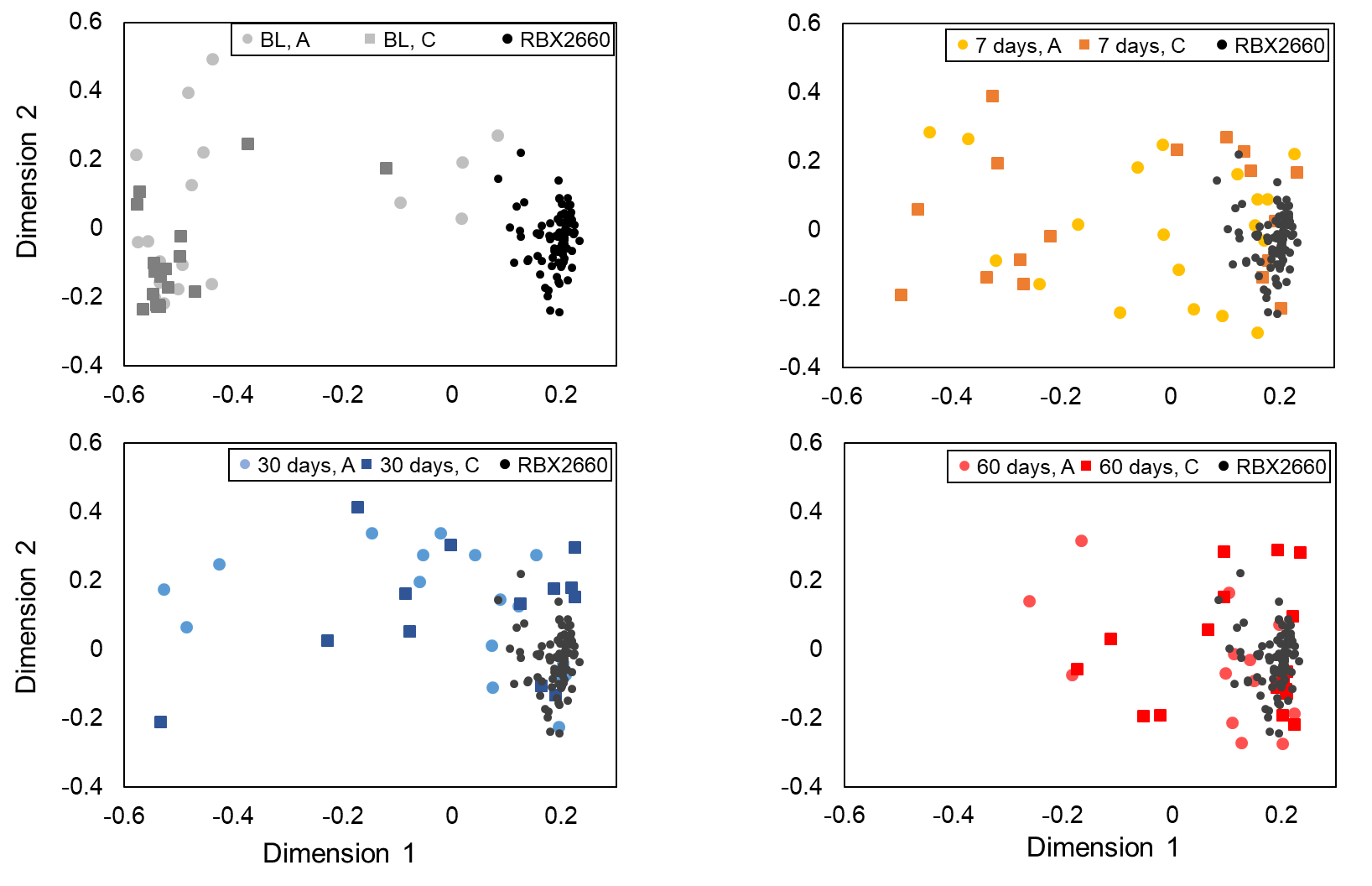


**Supplementary Figure S2.1**. Relative abundances of families within *Bacteroidia* class, expressed as a fraction of the total *Bacteroidia* class bacteria at each indicated time point.


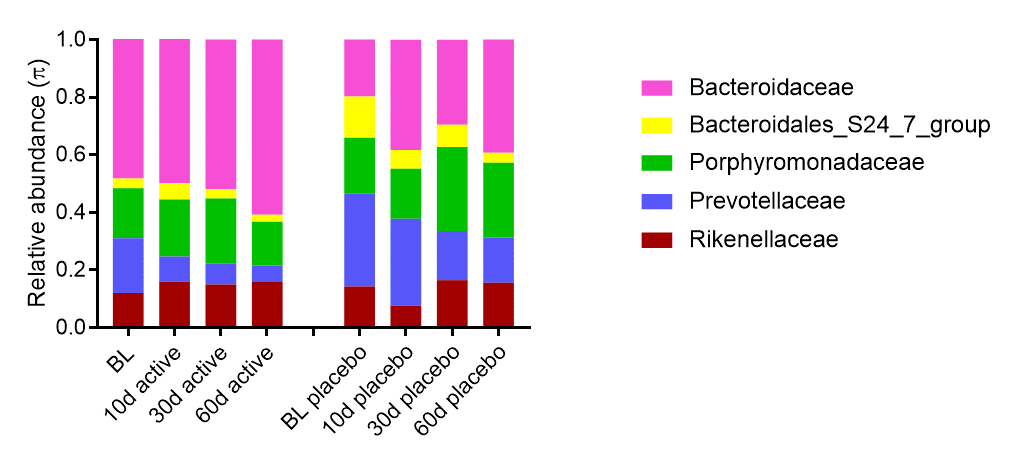


**Supplementary Figure S2.2**. Relative abundances of families within *Clostridia* class, expressed as a fraction of the total *Clostridia* class bacteria at each indicated time point.


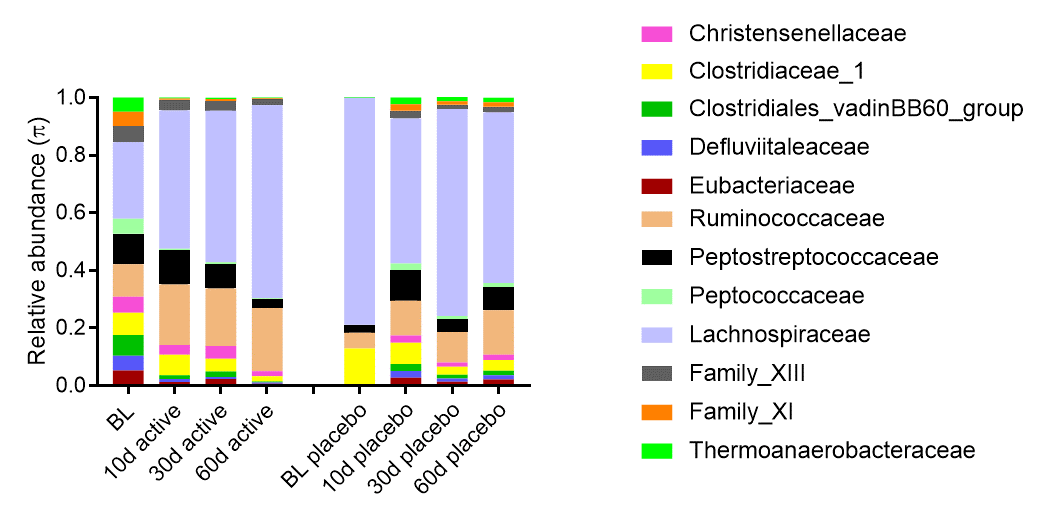


**Supplementary Figure S2.3**. Relative abundances of families within *Gammaproteobacteria* class, expressed as a fraction of the total *Gammaproteobacteria* class bacteria at each indicated time point.


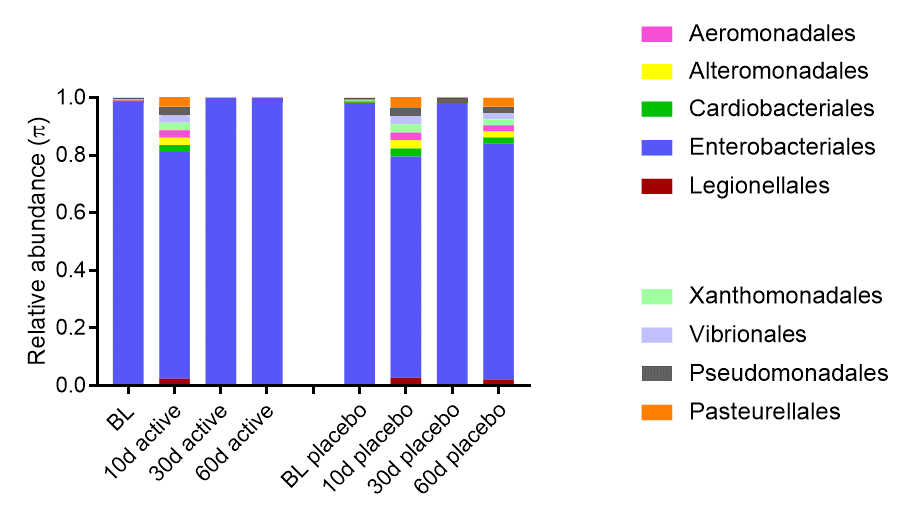


**Supplementary Figure S2.4**. Relative abundances of families within *Bacilli* class, expressed as a fraction of the total *Bacilli* class bacteria at each indicated time point.
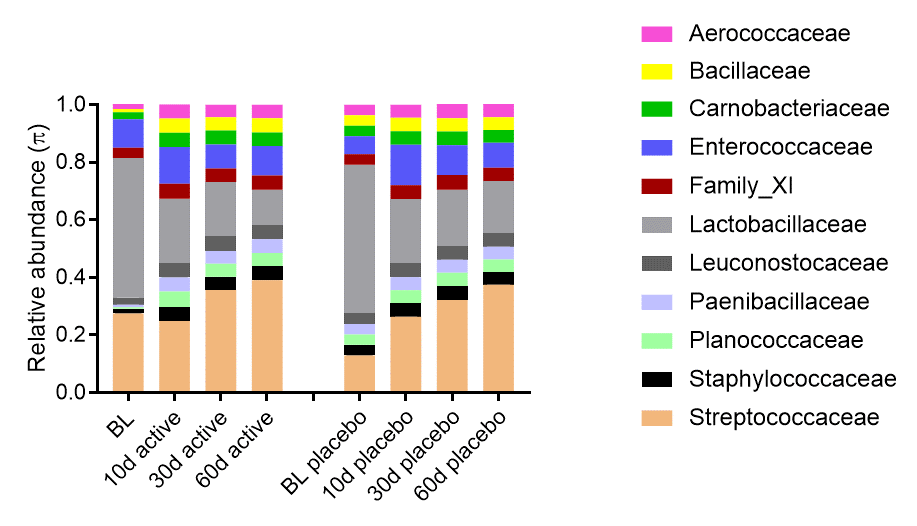


**Supplementary Table S1**. Comparison of relative taxonomic abundance (π) between Groups A and C for each time point group. There was no significant difference between Groups A and C at any time point, as determined by Wald-type test.

| Time point | *p* value  Group A vs Group C |
| --- | --- |
| Baseline | 0.47 |
| 10 days | 0.95 |
| 30 days | 0.87 |
| 60 days | 0.49 |

**Supplementary Figure S3**. Effect size (ES) of each time point group among placebo responders compared to RBX2660. Mean relative abundance π vectors at the taxonomic class level are shown with upper and lower confidence intervals as determined by fitting to a Dirichlet-multinomial distribution. ES is expressed as φ for each pairwise comparison.

**
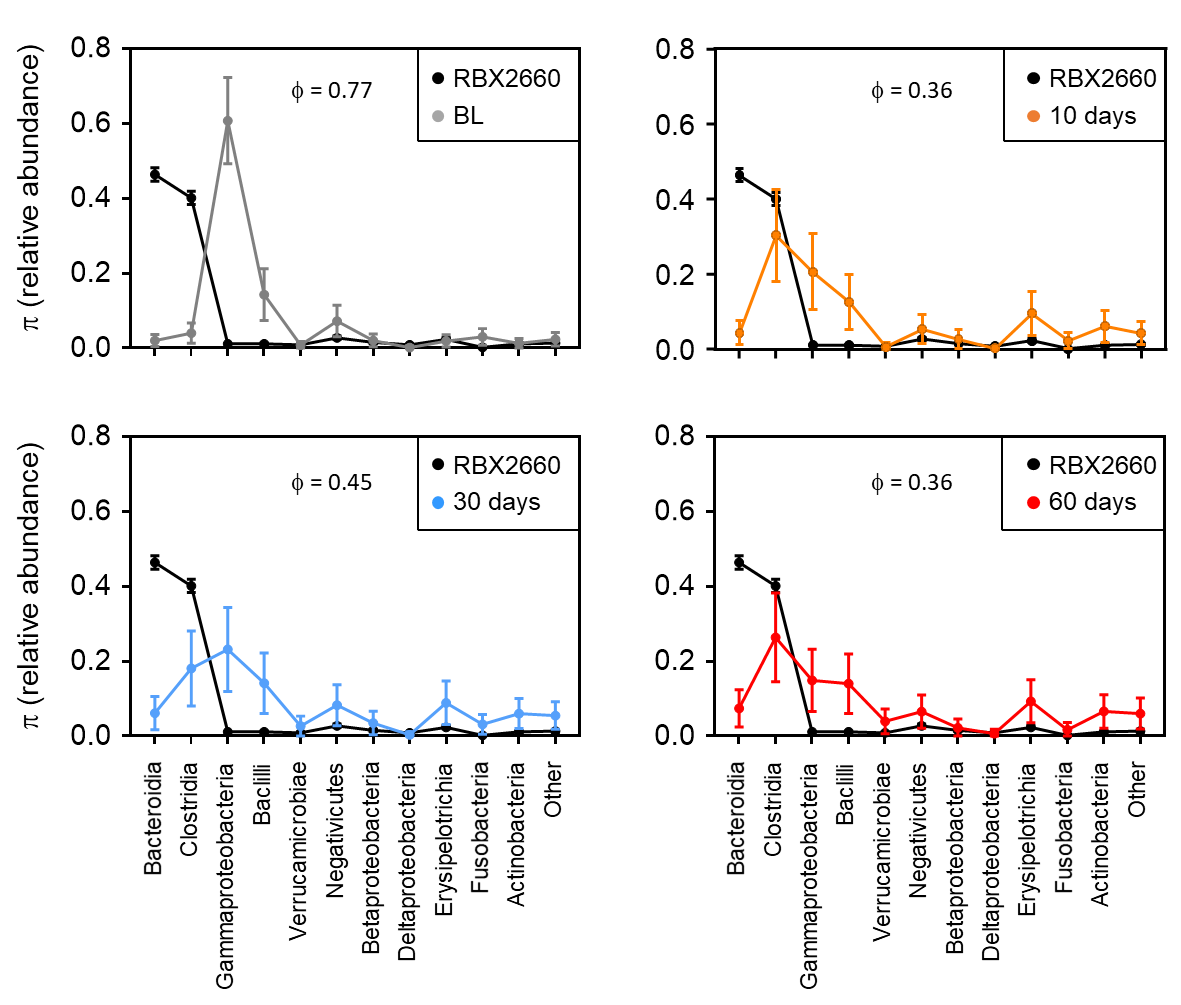
**

**Supplementary Table S2**. Overdispersion (θ) at order, family, and genus levels.

|  |  | **Order** | | **Family** | | **Genus** | |
| --- | --- | --- | --- | --- | --- | --- | --- |
|  | Group | Theta | Comparison to baseline (*p*) | Theta | Comparison to baseline (*p*) | Theta | Comparison to baseline (*p*) |
| RBX2660-treated | Baseline | 0.365 | NA | 0.343 | NA | 0.336 | NA |
|  | 7 days | 0.288 | .482 | 0.261 | .168 | 0.220 | <.0001 |
|  | 30 days | 0.260 | .101 | 0.230 | .018 | 0.186 | <.0001 |
|  | 60 days | 0.229 | <.0001 | 0.169 | <.0001 | 0.138 | <.0001 |
|  | Baseline | 0.261 | NA | 0.290 | NA | 0.409 | NA |
| Placebo-treated | 7 days | 0.346 | NA | 0.306 | .394 | 0.253 | <.0001 |
|  | 30 days | 0.329 | .030 | 0.277 | .041 | 0.193 | <.0001 |
|  | 60 days | 0.351 | .100 | 0.297 | .095 | 0.248 | <.0001 |

**Supplementary Figure S4**. Comparison of mean relative abundance data at the taxonomic class level for samples included in repeated measures analysis among RBX2660- or placebo-treated participants.


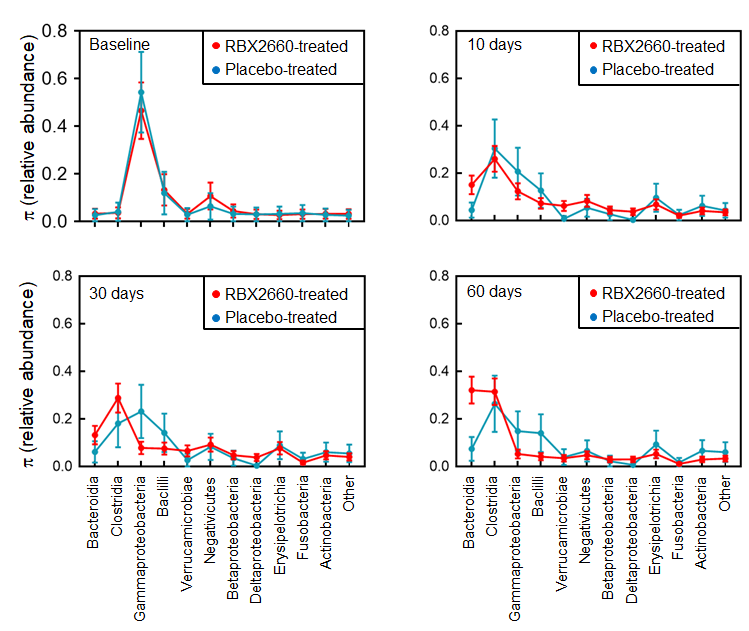


**Supplementary Table S3**. Relative abundance of key taxonomic classes at baseline among three trial participants administered fidaxomicin prior to treatment.

|  |  |  | **Relative Abundance** |  |
| --- | --- | --- | --- | --- |
| Participant ID | *Bacteroidia* | *Clostridia* | *Gammaproteobacteria* | *Bacilli* |
| 1 | 0.0027 | 0.8131 | 0.0103 | 0.0615 |
| 2 | 0.0378 | 0.0015 | 0.2064 | 0.1885 |
| 3 | 0.00031 | 0.0002 | 0.3091 | 0.6578 |

**REFERENCES**

1. Dubberke ER, Lee CH, Orenstein R, Khanna S, Hecht G, Gerding DN. Results From a Randomized, Placebo-Controlled Clinical Trial of a RBX2660-A Microbiota-Based Drug for the Prevention of Recurrent Clostridium difficile Infection. Clin Infect Dis **2018**; 67(8): 1198-204.

2. Human Microbiome Project C. Structure, function and diversity of the healthy human microbiome. Nature **2012**; 486(7402): 207-14.

3. Edgar RC. Search and clustering orders of magnitude faster than BLAST. Bioinformatics **2010**; 26(19): 2460-1.

4. Edgar RC. UPARSE: highly accurate OTU sequences from microbial amplicon reads. Nat Methods **2013**; 10(10): 996-8.

5. Pruesse E, Quast C, Knittel K, et al. SILVA: a comprehensive online resource for quality checked and aligned ribosomal RNA sequence data compatible with ARB. Nucleic Acids Res **2007**; 35(21): 7188-96.

6. La Rosa PS, Brooks JP, Deych E, et al. Hypothesis testing and power calculations for taxonomic-based human microbiome data. PLoS One **2012**; 7(12): e52078.

7. La Rosa PS, Zhou Y, Sodergren E, Weinstock G, Shannon WD. Hypothesis Testing of Metagenomic Data. In: Izard J, Rivera MC. Metagenomic for Microbiology: Academic Press, **2015**:81-96.

8. Cox TF, Cox MAA. Multidimensional scaling. 2nd ed. Boca Raton: Chapman & Hall/CRC, **2001**.

9. Legendre P, Legendre L. Numerical ecology. Third English edition. ed. Amsterdam: Elsevier, **2012**.
